# 402. COVID-19 Infection in Nepal: Epidemiological Analysis from April 2020 to March 2021

**DOI:** 10.1093/ofid/ofab466.603

**Published:** 2021-12-04

**Authors:** Hai V Le

**Affiliations:** Department of Orthopaedic Surgery, University of California, Davis, Elk Grove, California

## Abstract

**Background:**

In December 2019, SARS-CoV-2 or coronavirus disease 2019 (COVID-19) emerged from Wuhan, China. A global pandemic quickly unfolded, infecting >137 million people and causing >2.9 million deaths globally as of April 13, 2021. Before April 1, 2020, there were only five confirmed COVID-19 cases in Nepal. Like many countries around the world, the COVID-19 situation quickly escalated in Nepal. The purpose of this study was to determine the trends in COVID-19 cases and deaths in Nepal from April 2020 to March 2021.

**Methods:**

We utilized epidemiological data from daily Situation Reports published by the Ministry of Health and Population (MOHP) of Nepal. Data were extracted or calculated from April 1, 2020 to March 31, 2021. Primary variables of interest were national and provincial daily cases, total cases, daily deaths, and total deaths.

**Results:**

Between April 1, 2020 to March 31, 2021, there were 277,304 cases. October 2020 had the highest monthly cases with 92,926 cases. During the one-year study period, the infection rate was 915 cases per 100,000 people. The largest single-day new cases was October 21, 2020 with 5,743 cases, which is calculated to 19 cases per 100,000 people. There were a total of 3,030 deaths. The largest daily new deaths was November 4, 2020 with 43 cases. June 10, 2020 had the highest number of people in quarantine with 172,266 people. October 23, 2020 had the highest number of active cases with 46,329 cases. By March 31, 2021, the percent of mortality was 1.1%, active infection was 0.5%, and recovery was 98.4%.

**Conclusion:**

Nepal had lower COVID-19 infection and case-fatality rates compared to other countries most affected by the pandemic. This was due to several factors, most notably early implementation of strict lockdown measures and closing of international borders on March 24, 2020 after the second confirmed COVID-19 case. As lockdown restrictions were lifted on July 7, 2020, COVID-19 cases and deaths in Nepal rose rapidly. As vaccination begun on January 27, 2021, cases started to slow down until the most recent outbreak coinciding with the second wave in its neighboring country, India. Now, infection and case-fatality rates in Nepal are at an all-time high, prompting further lockdowns on April 29, 2021.

**Disclosures:**

**All Authors**: No reported disclosures

